# A systematic study on the influence of thermodynamic asymmetry of 5′-ends of siRNA duplexes in relation to their silencing potency

**DOI:** 10.1038/s41598-018-36620-9

**Published:** 2019-02-21

**Authors:** Jolanta Lisowiec-Wąchnicka, Natalia Bartyś, Anna Pasternak

**Affiliations:** Department of Nucleic Acids Bioengineering, Institute of Bioorganic Chemistry, Polish Academy of Sciences, Noskowskiego 12/14, 61-704 Poznan, Poland

## Abstract

siRNA molecules possess high potential as molecular tools and can be used as effective therapeutics in humans. One of the key steps in the action of these molecules is the choice of antisense strand by the RNA-induced silencing complex (RISC). To explain this process, we verified the theory which states that antisense strand selection is based on the thermodynamically less stable 5′ end of siRNA. Based on the studies presented herein, we observed that for the tested siRNA duplexes, the difference in the thermodynamic stability of the terminal, penultimate and pre-penultimate pairs in the duplex siRNA is not the dominant factor in antisense strand selection. We found that both strands in each tested siRNA molecule are used as an antisense strand. The introduction of modified nucleotides, whose impact on the thermodynamic stability of siRNA duplexes was studied, results in changes in antisense strand selection by the RISC complex. The presence of a modified residue often caused predominant selection of only one antisense strand which is at variance with the theory of siRNA strand bias.

## Introduction

The control of genetic information expression *via* RNA interference was discovered in 1998 by Fire *et al*. in the model organism *Caenorhabditis elegans*^1^. Subsequently, RNA interference (RNAi) mechanisms have also been discovered in mammalian cells^2^. These findings have led to the design of artificial siRNA molecules that can be introduced into cells and allows the study of the regulation of gene expression, the function of different genes and the relationship between gene products^3^. In addition, the development of RNAi technology has identified the gene responsible for cancer diseases^4^. High selectivity, relatively cheap synthesis, and ease of chemical modification are the main features that determine siRNAs as candidates for new generation therapeutics. Naturally occurring siRNAs mainly originate from environmental RNAs or from editing of the host transcript. The first siRNA molecules were observed as defense products against viruses or transgenes^5^. Subsequently, it was discovered that siRNAs can be derived from centromeres, transposons and other repeating sequences^6^, as well as from specific genomic transcripts^7,8^. siRNA molecules are formed by long double-stranded RNA fragments that are cleaved by Drosha nuclease to the long RNA hairpins^9^. Then, the Dicer nuclease cleaves these molecules to mature siRNAs that can bind to the RISC proteins forming the pre-RISC complex. A mature siRNA molecule consists of two 21–23 nucleotide strands. The ~19 nt long fragment forms a duplex, while two nucleotides on each oligonucleotide 3′-end remain unhybridized and form two 3′-overhangs^10–12^. The mature siRNA molecule interacts with the RNA-induced silencing complex (RISC), and one strand is selected from the duplex as an antisense strand by the Ago protein. Directly afterwards, the active RISC complex binds to the mRNA fragment, which is complementary to the antisense strand, the mRNA is degraded and the expression of the gene is blocked^13,14^.

Technology based on siRNA molecules can be divided into two approaches: (a) achieving gene silencing by introducing into the cell a plasmid coding sequence of long RNA hairpins that are edited in the cell to mature siRNAs; or (b) introducing mature, artificial siRNAs. The future potential for introducing mature forms seems to be more appropriate for the conception of siRNA-based therapies because of the possibility of the chemical modification of oligonucleotides. The number of studies on siRNA based strategies is increasing but simultaneously the number of reports on the use of siRNA as a drug is decreasing^15^. This is due to problems with the penetration of siRNAs through the cell membrane, off-target effects, and the fact that siRNA molecules may be responsible for immune response^16^. Nevertheless, it is possible, similar to the case of antisense oligonucleotides, that chemical modification of nucleotide residues or structural modifications will allow the development of siRNAs with attractive therapeutic properties.

One of the causes of off-target effects is silencing of non-target genes based on the sense-strand-mediated pathway^17^. Each of the two strands in the siRNA duplex has the potential to be an antisense strand^18^. Studies on the selection of antisense strand by the RISC protein in a *Drosophila* model organism showed that the process is based on the thermodynamic asymmetry of the ends of the siRNA duplex and the detection of the strand bias occurs *via* the RISC loading complex (RLC). In *Drosophila*, Dicer2 and R2D2 proteins recognize the thermodynamic asymmetry of the siRNA molecule, which causes Dicer2 to bind to the less stable 5′-end, whereas the R2D2 protein interacts with the more stable 5′-end of siRNA^19–21^. This mechanism promotes the strand with a less stable 5′-end as the antisense one. In humans, the Ago2 protein alone can select an antisense strand; however, other proteins (Dicer and TRBP or PACT) have also been identified as having an influence on strand selection in the RISC complex^22,23^. Moreover, it seems that various factors such as 5′-end thermodynamics, sequence of siRNA ends, and structural features may affect guide strand selection efficiency in humans^23^.

Here, we present comprehensive thermodynamic and biological activity studies of siRNAs with defined thermodynamic stability of the ends. We have developed a method for the quantitative evaluation of the use of both strands by the RISC complex, using the HeLa cell line and a qPCR reaction. In addition, the modified nucleotides, *i*.*e*. 2-thiocytidine, 2-thiouridine, and 4-thiouridine, and mismatches were introduced in terminal, penultimate and pre-penultimate position of model siRNA molecules. Using the UV melting method, we determined the effect of these changes on the thermodynamic stability of model duplexes. Subsequently, modified nucleotides and mismatches were introduced in the selected positions of full length siRNA duplexes to examine their effect on strand selection by the RISC complex in mammalian HeLa cell line.

## Results and Discussion

### Antisense strand selection in siRNA duplexes with defined thermodynamic stability of the ends

**Figure 1:**
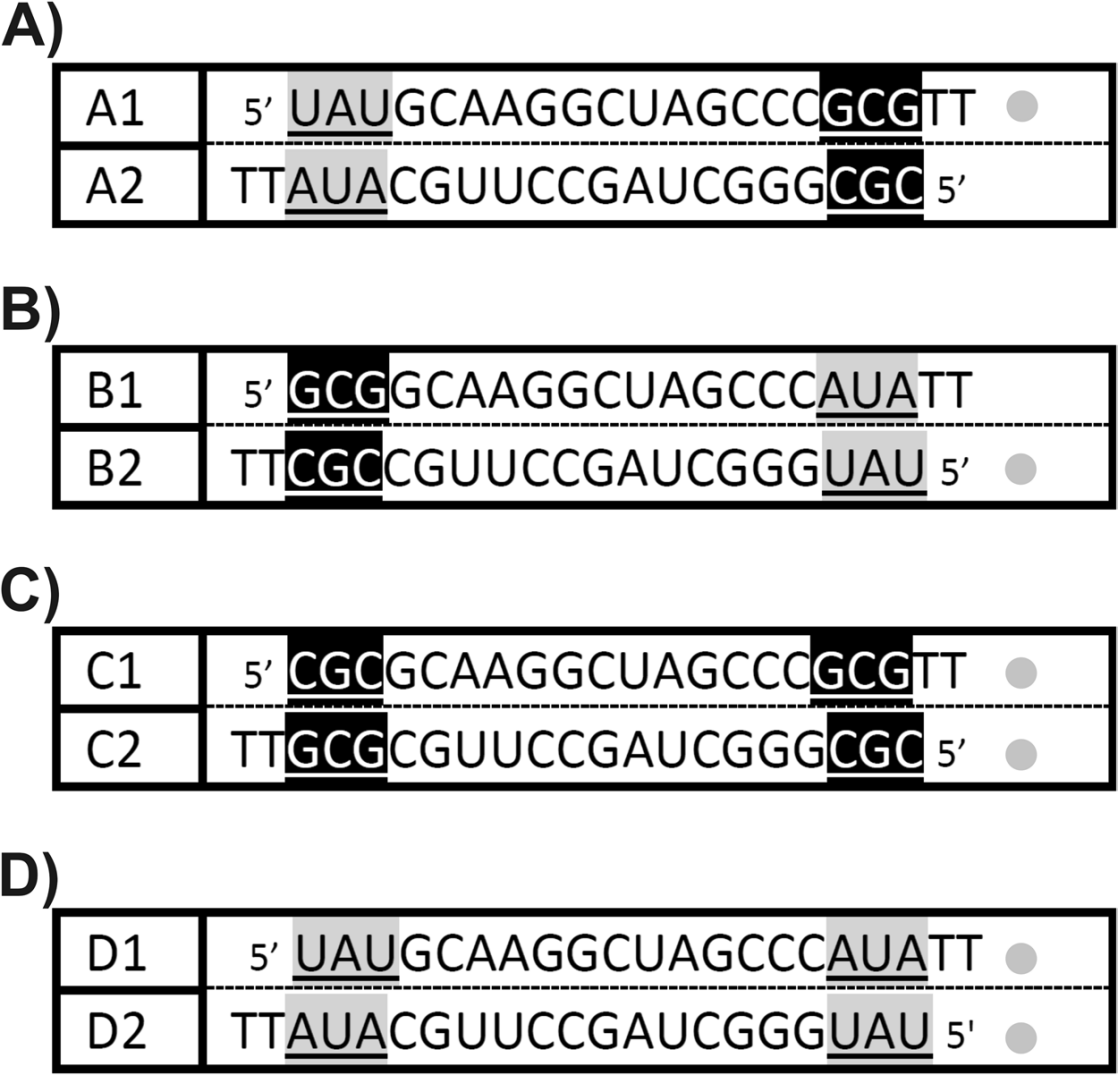
Sequences of siRNA duplexes with defined 5′-end thermodynamic stability. The duplexes with thermodynamically diversified 5′ ends are shown in panels (A) A1/A2 and (B) B1/B2. The duplexes with equal thermodynamic stability of 5′ ends are shown in panels (C) C1/C2 and (D) D1/D2. A dot near a specific strand indicates the strand which should be selected as antisense strand according to the theory of a less stable 5′-end.

**Figure 2:**
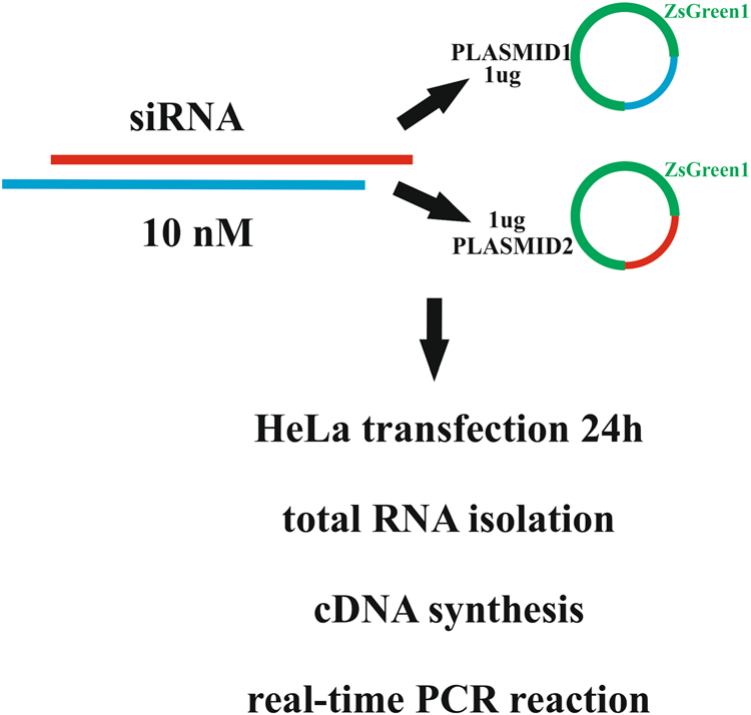
Scheme of HeLa cell line experiments. Each siRNA duplex was examined in two individual experiments. The siRNA molecule was co-transfected with two plasmids. One of these contained an insert with a complementary sequence to one of the siRNA strands, while the second plasmid contained an insert with a complementary sequence to the second strand of the siRNA duplex. After 24 h total RNA isolation, cDNA synthesis and qPCR analysis were performed.

**Figure 3:**
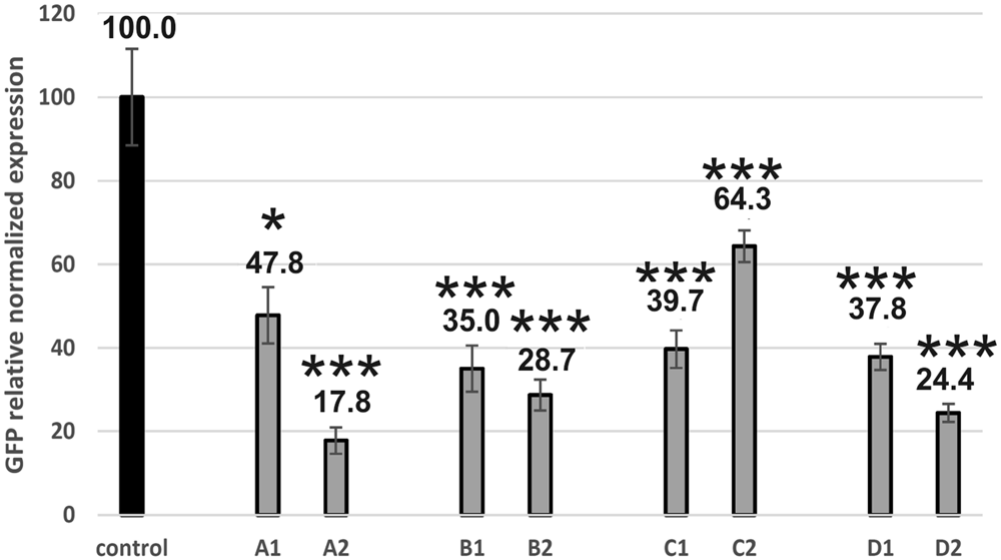
The change of ZsGreen1 relative normalized expression induced by siRNAs: A1/A2, B1/B2, C1/C2, D1/D2. Each bar represents the mean value ± standard deviation (SD) of n = 3–5. *Indicates statistical significance at a level of P < 0.05; ***indicates statistical significance at a level of P < 0.01.

Four main siRNA molecules with defined thermodynamic stability of the ends were designed to evaluate the antisense strand selection by the RISC complex (Fig. 1). All designed siRNAs have an identical core sequence and differ only in the sequence of the last three base pairs. The A1/A2 molecule has one duplex end that is less thermodynamically stable, while the other has higher thermodynamic stability (Fig. 1A). In the second duplex of the tested siRNAs, *i*.*e*. B1/B2, the situation is reversed but the difference in stability remains the same (Fig. 1B). The difference in the thermodynamic stability between the ends in duplexes A1/A2 and B1/B2 is 3.97 kcal/mol, as calculated based on nearest neighbor parameters^24^. The third duplex, C1/C2, has two ends of comparable thermodynamic stability (Fig. 1C). The last tested duplex, D1/D2, contains two ends with lower thermodynamic stability in the last three duplex pairs; nevertheless, the stability of both ends is also the same (Fig. 1D). To investigate the antisense strand selection by the RISC complex, the system was developed in such a way that allows quantitative detection of mRNA levels of ZsGreen1 proteins and assessment of ZsGreen1 gene expression. Each of the siRNA duplexes was tested in two ways (Fig. 2). First, the co-transfection of the siRNA molecule and the plasmid into the HeLa cell line was performed. The complementary fragment of one siRNA strand was cloned to the plasmid. Then, the same siRNA molecule was co-transfected with another plasmid containing the fragment which was complementary to the second strand. Calculation of the quantitative expression of the mRNA of the ZsGreen1 gene allowed the evaluation of the selection of individual strands by the RISC as the antisense strands. According to the theory of antisense strand selection during RNA interference, the antisense strand is selected based on the thermodynamic asymmetry of the ends. The strand with the less stable 5′-end is chosen as the guide strand. In addition, a difference of only 0.5 kcal/mol in stability of the 5′-ends is sufficient for such selection^19,20^. According to this rule, in an siRNA A1/A2 duplex, the A1 strand should mainly be selected as the antisense strand, whereas in the B1/B2 duplex the B2 strand should be selected as the guide strand. For duplexes with the same stability of the ends (duplexes C1/C2 and D1/D2), strand selection should be at comparable levels. Only for the C1/C2 duplex could the silencing of the ZsGreen1 gene be less efficient, and this is due to the relatively high thermodynamic stability of the siRNA ends and therefore due to difficulties in the unfolding of the helix by the RISC complex proteins^25^. The results of a quantitative analysis of the ZsGreen1 gene transcript by the qPCR indicate that for the A1/A2 duplex both strands can be used as the antisense strand. However, in contrast to the theory of the less stable 5′-end, the A2 strand is more widely used as the guide strand and this reduces the expression of the ZsGreen1 mRNA by 82.2%, while the A1 strand decreases the expression by 52.2% relative to the control (Fig. 3). In the second duplex, B1/B2, despite the thermodynamic asymmetry of the ends, the silencing the ZsGreen1 gene is similar for the two strands, and the ZsGreen1 mRNA level relative to the control reaches 35% for B1 and 28.7% for B2 (Fig. 3). The quantitative analysis of qPCR confirmed that in the case of a duplex with thermodynamically equivalent ends containing G-C base pairs, both strands can be selected as guide strand; however, the choice between strands C1 and C2 is not the same (Fig. 3). The C1 strand decreases the expression of the ZsGreen1 gene mRNA by 60.3% and the C2 strand by 35.7%. A duplex with thermodynamically equivalent ends containing A-U base pairs causes a down-regulation of the ZsGreen1 gene mRNA: 62.2% for D1, and 75.6% for D2 (Fig. 3). The above results clearly show that in the HeLa cell line, the choice of antisense strand is not consistent with the theory that the strand with the less stable 5′ end is selected as the antisense strand. The first studies on this issue were made using proteins from a *Drosophila* model organism where it was shown that the Dicer and R2D2 proteins are involved in the selection of the antisense strand based on the difference in thermodynamic stability of the siRNA ends^19–21^. There is evidence that in the human RISC, the Ago2 protein itself can select the guide strand, whereas Dicer and TRBR or PACT proteins help in this mechanism^22,23^. The MID domain in the Ago2 protein is responsible for the selection of the antisense strand, because in the mature RISC, where one strand remains, this domain interacts with the 5′ end of the strand^26^. An exact explanation of the antisense strand selection process seems to be difficult due to the lack of the solved tertiary structure of the full-length human Ago2 protein and the pre-RISC complex where both strands are found^26^. Our research confirms that in the human RISC, thermodynamic stability of siRNA ends is not the only factor that determines antisense strand selection and that other factors exist, such as siRNA sequence or structure and the thermodynamic stability of the ends^23^. However, if siRNA molecules are considered active when there is a decrease in expression of at least 50%, then only C2 as an antisense strand does not meet these criteria. Of course, in the task of silencing a particular gene or evaluating the effectiveness of certain siRNA molecules, researchers only study the effect of the strand that is considered to be the antisense one. In the presented studies, although we recognize an siRNA molecule as active, a situation may occur wherein the passenger strand is chosen by the RISC complex more often than the one that is considered the guide strand. Such a situation can lead to off-target effects based on sense strand pathway mechanisms^17^. In addition, despite the design of siRNA molecules with thermodynamically asymmetric ends from randomly chosen siRNA molecules, only 58–78% of such molecules show efficacy at more than 50% and only 11–18% at 90–95%, which also indicates that 5′-end thermodynamic asymmetry is not a key factor in the selection of the antisense strand^27^. It seems that in RNAi technology, which could become a modern therapeutic approach for humans, situations in which two strands are selected as an antisense strand should be excluded. This disadvantage leads to the already mentioned off-target mechanism and may lead to the need for a higher concentration of siRNA molecules for effective gene silencing. In addition, RISC proteins are involved in interactions with the non-active siRNA molecules, which might influence other cell pathways because the same RISC proteins are used in miRNA interference mechanisms^28–30^.

### The effect of modified nucleotides and mismatches on the thermodynamic stability of model siRNA duplexes

**Figure 4:**
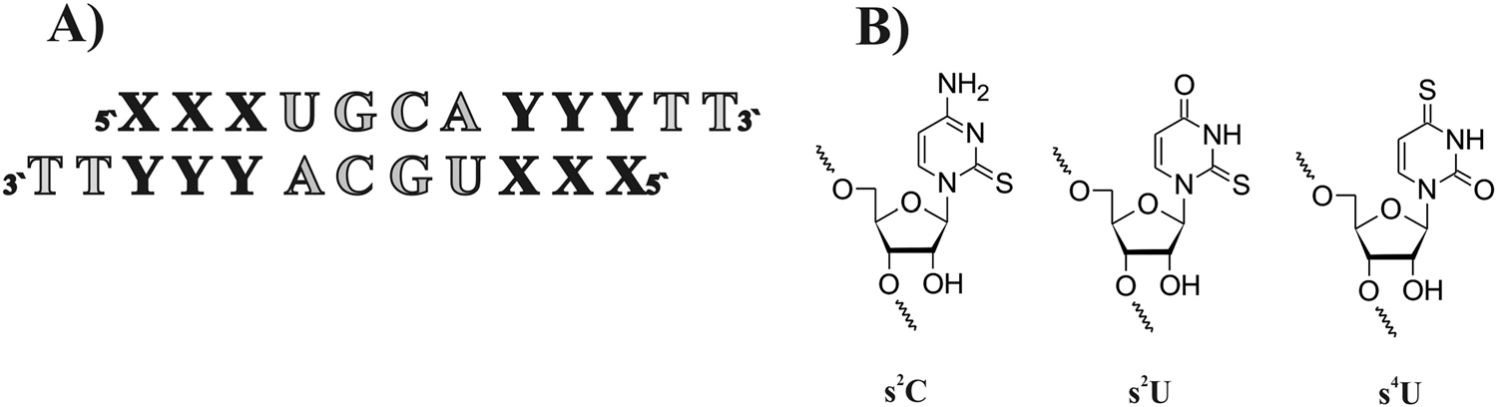
Schematic representation of siRNA duplexes and modified nucleosides used in the thermodynamic studies: (**A**) Model siRNA duplex used in UV melting experiments, X-Y correspond to unmodified or modified base pairs or mismatches, (**B**) structure of 2-thiocytidine, 2-thiouridine, and 4-thiouridine.

**Figure 5:**
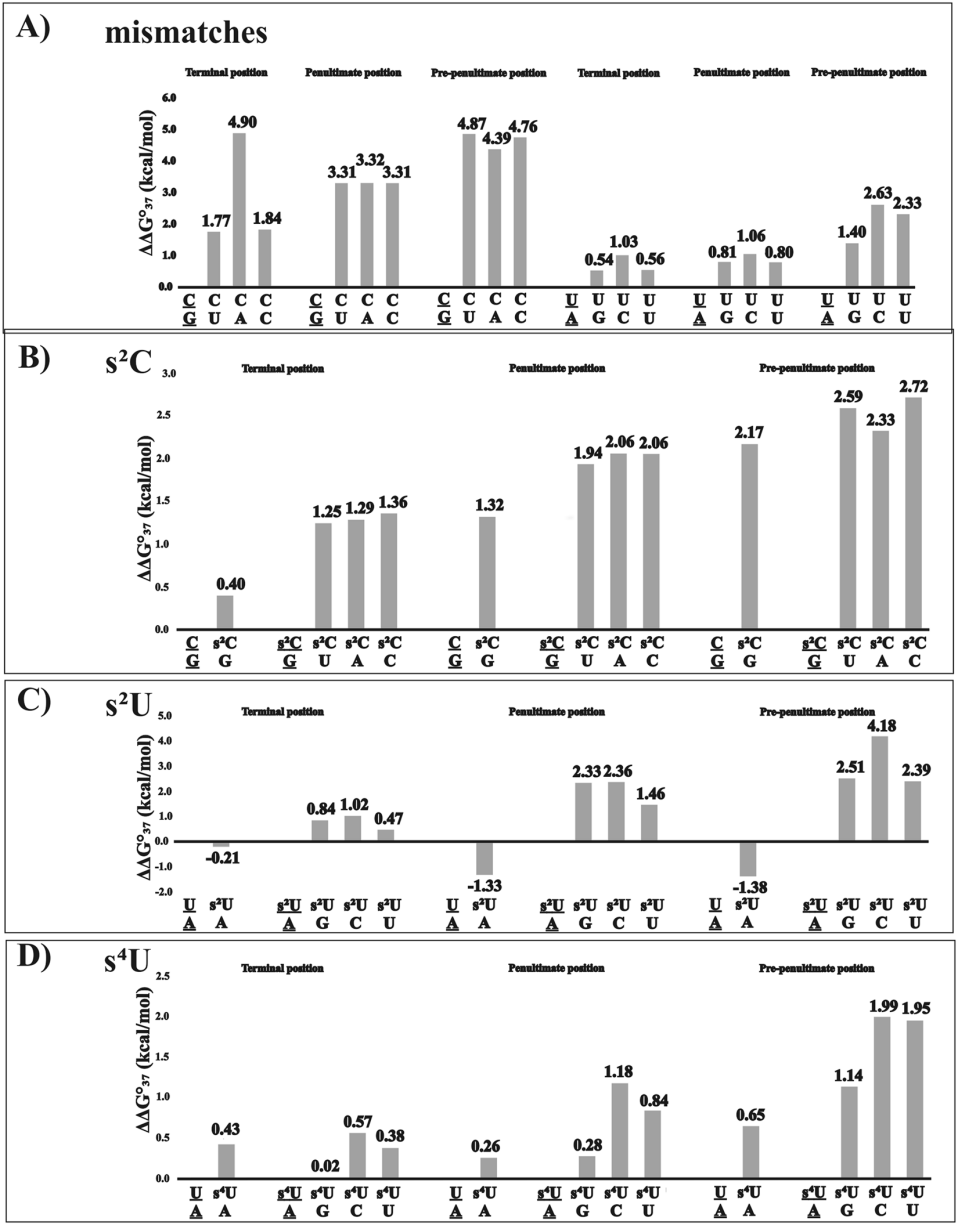
Results of UV melting experiments. ΔΔG°_37_ corresponds to the difference in free energy between a model duplex and a duplex containing a mismatch or modified nucleotide. Positive bar values indicate thermodynamic destabilization and negative bar values indicate thermodynamic stabilization. Underlined base pairs mean references for which a free energy difference was determined. (**A**) The influence of mismatches (**B**) the influence of s^2^C substitution, (**C**) the influence of s^2^U substitution, (**D**) the influence of s^4^U substitution.

Many chemical modifications have so far been tested to improve the potential and effectiveness of siRNAs. Thionucleosides are well-known as chemically modified ribonucleosides that naturally occur in anticodon loops of many tRNAs^31–33^. The presence of sulfur instead of oxygen in the carbonyl group of pyrimidines may increase base pairing specificity or decrease mismatch discrimination^34^. The aim of this part of our studies was to analyze the influence of the most commonly known thionucleosides on siRNA strand bias. In order to investigate the effect of 2-thiocytidine (s^2^C), 2-thiouridine (s^2^U) and 4-thiouridine (s^4^U) on the thermodynamic stability of siRNA duplexes, we designed model RNA duplexes with 3′TT dangling ends (Fig. 4). The oligonucleotides were designed to mimic siRNA duplexes, *i*.*e*. they consisted of an RNA core with 3′TT overhangs. The sequences were self-complementary, which resulted in lower errors in the s^2^C, s^2^U or s^4^U contributions^35^. The actual ΔΔG°_37_ values were divided by two to derive the single modification effect (Fig. 5; detailed thermodynamic parameters are included in the Supplementary File, Tables S1, S2, S3). The modified nucleosides were introduced at the last three terminal positions of the RNA helix for detailed analysis of their influence on the relative stability of siRNA 5′-ends in relation to gene silencing efficiency (Fig. 5).

**Figure 6:**
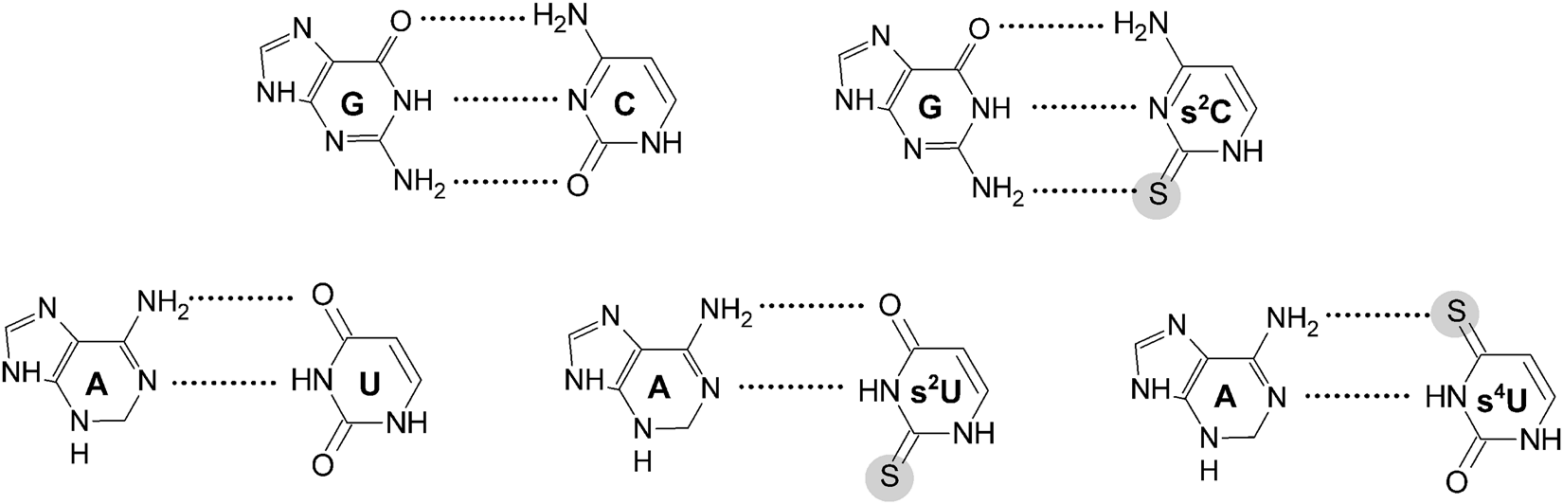
Hydrogen bonding between G-C, G-s^2^C, A-U, A-s^2^U, and A-s^4^U.

Introduction of an s^2^C residue at 5′-terminal position causes destabilization by 0.40 kcal/mol in relation to an unmodified siRNA duplex of the same sequence. As expected, the unfavorable thermodynamic effect increases when s^2^C is shifted toward the helix center, and the destabilization reaches 1.32 and 2.17 kcal/mol for penultimate and pre-penultimate positions, respectively (Fig. 5B). The stacking preferences are similar for C and s^2^C^36^. On the other hand, sulfur is less electronegative than oxygen; therefore, it forms a weaker hydrogen bond. The destabilization induced by the presence of s^2^C might be due to the disruption of hydrogen bonding between the amino group of guanine and the thiocarbonyl of s^2^C (Fig. 6). The van der Waals radius of a carbonyl is 0.45 Å shorter in comparison to that of a thiocarbonyl; thus, the presence of sulfur in s^2^C might cause some steric clashes when interacting with guanosine^36,37^. The increased destabilization which is observed when moving toward siRNA central positions remains in accordance with many other articles indicating that incorporation of modified nucleosides internally contributes more to the overall thermodynamic stability of the helix in reference to terminal position^38–41^.

The analysis of siRNAs containing mismatches indicates that s^2^C significantly decreases mismatch discrimination (Fig. 5B). The presence of s^2^C-U, s^2^C-A and s^2^C-C mismatches destabilizes duplexes thermodynamic stability relative to siRNAs containing s^2^C-G base pairs by a similar magnitude when incorporated at the same position, *i*.*e*. 1.25–1.36 kcal/mol for mismatches at the 5′-end, 1.94–2.06 kcal/mol at penultimate position and 2.33–2.72 kcal/mol at pre-penultimate position. In comparison, C-U, C-A and C-C mismatches placed at the same positions lead to decreases in duplex thermodynamic stability: 1.77–4.90, 3.31–3.32 and 4.39–4.87 kcal/mol for 5′-end, penultimate and pre-penultimate positions, respectively (Fig. 6A). Such decreases in mismatch discrimination in the presence of s^2^C is in agreement with previous observations that the thiocarbonyl group causes some disruption of hydrogen bonding in s^2^C-G base pairs. Interactions of s^2^C with U, A or C residues cause lower net effects in comparison to those induced by cytidine. The hydrogen bond between the carbonyl and amino groups in C-G base pairs is strong and loss of these kinds of interactions due to another H-bonding pattern observed in C-U, C-A or C-C mismatches causes more apparent energetic consequences in terms of s^2^C-G base pair and s^2^C mismatches.

In contrast, interactions of the s^2^U residue with adenosine (s^2^U-A base pair) are favorable for siRNA duplex stability (Fig. 5C). Although s^2^U at the 5′-end causes stabilization by only 0.21 kcal/mol, the favorable thermodynamic effect significantly increases to 1.33 and 1.38 kcal/mol when shifting toward internal positions, *i*.*e*. penultimate and pre-penultimate, respectively. Hydrogen bonding in s^2^U-A base pairs does not involve thiocarbonyl groups (Fig. 6), but the presence of more polarizable sulfur might increase stacking interactions, which is obviously less pronounced at the duplex 5′-end. Moreover, it was previously reported that the presence of less electronegative sulfur at C2 increases N3 proton acidity and improves its ability to form hydrogen bonding with the N1 of adenosine^42,43^.

The presence of s^2^U mismatches at the siRNA 5′-end decreases duplex thermodynamic stability by 0.84, 1.02 and 0.47 kcal/mol for s^2^U-G, s^2^U-C and s^2^U-U, respectively, relative to siRNA containing an s^2^U-A base pair (Fig. 5C). What is more, this unfavorable effect is usually less pronounced in unmodified siRNA series. Those duplexes with terminal U-G, U-C and U-U mismatches are less stable in comparison to fully matched siRNAs: by 0.54, 1.03, and 0.56 kcal/mol, respectively. This increased mismatch discrimination is observed for internal s^2^U interactions relative to canonical mismatches. The s^2^U mismatches placed at penultimate position destabilize siRNA duplexes by 2.33 (s^2^U-G), 2.36 (s^2^U-C), and 1.46 kcal/mol (s^2^U-U) relative to duplexes with an s^2^U-A base pair, and at pre-penultimate position by 2.51 (s^2^U-G), 4.18 (s^2^U-C), and 2.39 kcal/mol (s^2^U-U). In contrast, natural mismatches decrease thermodynamic stability of siRNA duplexes by only 0.82 (U-G), 1.06 (U-C), and 0.80 kcal/mol (U-U) at penultimate position and by 1.40 (U-G), 2.63 (U-C), and 2.33 kcal/mol (U-U) at pre-penultimate position. The increased mismatch discrimination, and therefore higher base pairing specificity of s^2^U residues is due to the presence of a thiocarbonyl group. As already mentioned, the sulfur at position C2 is not involved in hydrogen bonding within s^2^U-A base pairs, but in the case of mismatches it might form a weak hydrogen bond with opposite nucleobases, increasing the destabilization effect in comparison to regular carbonyl groups at C2 of uridine.

As expected, the incorporation of 4-thiouridine is clearly unfavorable for the thermodynamic stability of siRNA duplexes (ΔΔG°_37_ = 0.26–0.65 kcal/mol per single s^4^U) (Fig. 5D). Our results are in accordance with previous reports^34^. The unfavorable thermodynamic effect is most probably due to the formation of weaker hydrogen bonds between less electronegative sulfur within thiocarbonyl of s^4^U and the amino group of adenosine (Fig. 6). As for s^2^C, the presence of s^4^U also decreases base pairing specificity. The presence of s^4^U-G, s^4^U-C and s^4^U-U mismatches at the 5′-end of siRNAs destabilize the duplex in the range of 0.02–0.57 kcal/mol in comparison to siRNAs containing s^4^U-A base pairs. The same type of mismatches formed with uridine decreases thermodynamic stability by 0.54–1.03 kcal/mol. A similar trend is observed at penultimate and pre-penultimate positions; however, the destabilization effect is usually larger relative to terminal positioning. The decrease in siRNAs thermodynamic stability induced by the presence of s^4^U mismatches placed at penultimate position is in the range of 0.28–1.18 kcal/mol, whereas shifting s^4^U mismatches toward the siRNA center causes destabilization by 1.14–1.99 kcal/mol (Fig. 5D). Analysis of the influence of regular U-G, U-C and U-U mismatches indicates that s^4^U at internal positions is also characterized by decreased mismatch discrimination, *i*.*e*. s^4^U mismatches usually destabilize less than U mismatches relative to siRNAs with s^4^U-A and U-A base pairs, respectively. Our data also are in accordance with previously published results indicating U-G (or s^4^U-G) mismatch as being most stable due to the lack of hydrogen bonding between sulfur and guanosine^34^. However, replacement of carbonyl by a thiocarbonyl group at C4 of uridine did not result in any stabilization of the s^4^U-G wobble base pair in relation to s^4^U-A, as had previously been reported^34^.

### The effect of modified nucleotides and mismatches on RISC-mediated antisense strand selection

**Figure 7:**
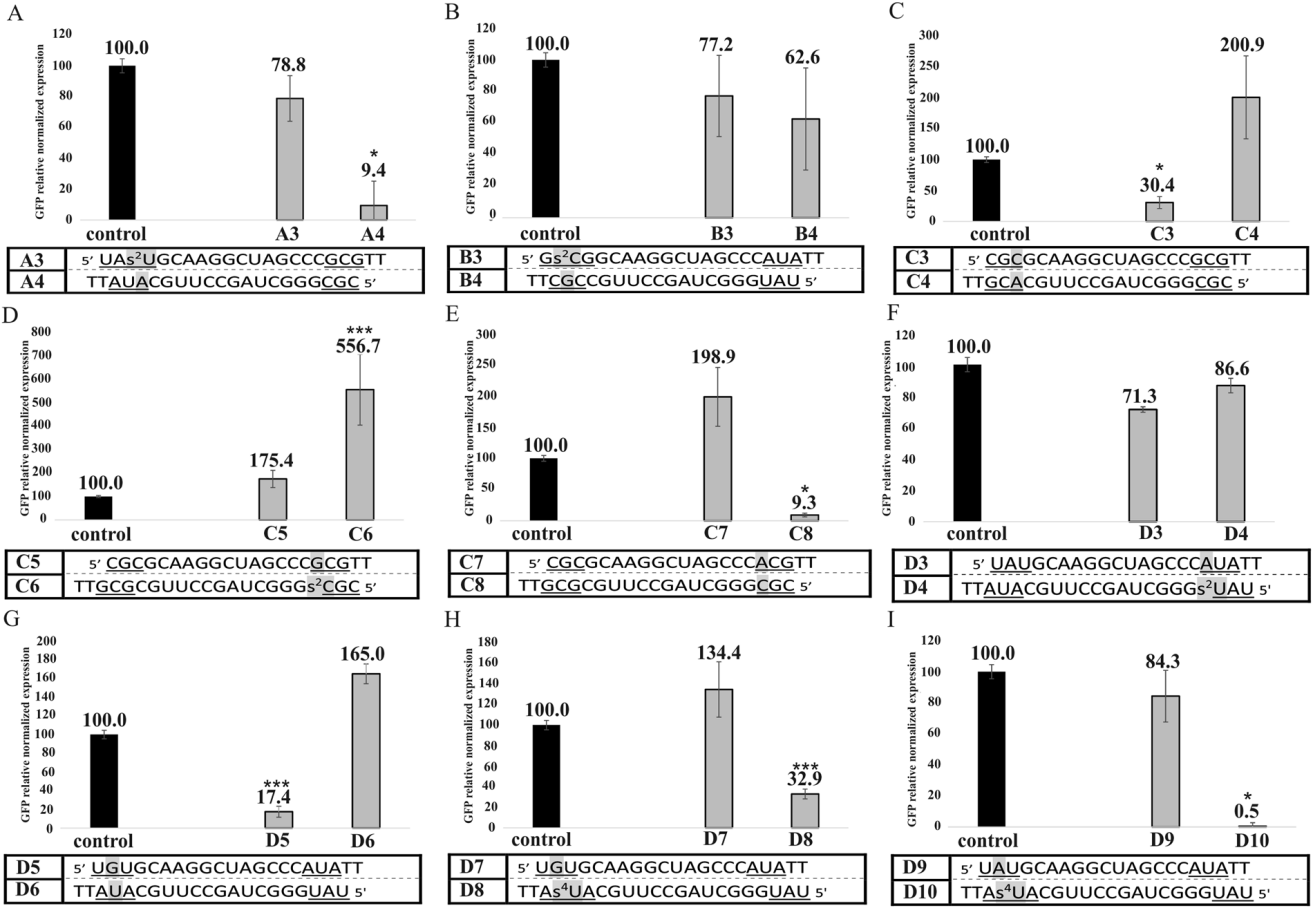
The change of ZsGreen1 relative normalized expression induced by siRNA molecules containing modified nucleotides. The activity of: (**A**) A3/A4 duplex with 2-thiouridine (s^2^U) at pre-penultimate position of A3 strand, (**B**) B3/B4 duplex with 2-thiocytidine (s^2^C) at penultimate position of B3 strand, (**C**) C3/C4 duplex with mismatch C-A at pre-penultimate position of C3 strand, (**D**) C5/C6 duplex with 2-thiocytidine (s^2^C) at pre-penultimate position of C6 strand, (**E**) C7/C8 duplex with mismatch C-A at pre-penultimate position of C7 strand, (**F**) D3/D4 duplex with 2-thiouridine (s^2^U) at pre-penultimate position of D4 strand, (**G**) D5/D6 duplex with mismatch G-U at penultimate position of D5 strand, (**H**) D7/D8 duplex with s^4^U-G mismatch at penultimate position of D8 strand, and (**I**) D9/D10 duplex with 4-thiouridine (s^4^U) at penultimate position of D10 strand. Each bar represents the mean ± standard deviation (SD) of n = 3–5. *Indicates statistical significance at a level of P < 0.05; ***indicates statistical significance at a level of P < 0.01.

Based on the UV melting experiments of siRNA model duplexes, the stabilizing or destabilizing effects of selected modified nucleotides and mismatches were demonstrated. We decided to introduce these changes into full length siRNA molecules and investigate their effect on the antisense and sense strand selection in HeLa cell lines. In addition, MTT assays were performed on all siRNA molecules tested on HeLa cell lines. These studies showed that under the experimental conditions (10 nM concentration of siRNA), these molecules are not toxic to HeLa cells and do not affect their growth (Table S4, Supplementary Information). The strand selection experiments were performed analogously to the studies on unmodified siRNA duplexes with defined thermodynamic stability of the ends. 2-Thiouridine (s^2^U) was introduced into the A3/A4 duplex at pre-penultimate position. Our thermodynamic analysis indicates that this change results in an increase in siRNA thermodynamic stability of 1.38 kcal/mol. Considering the large difference in thermodynamic stability of 5′-ends of unmodified duplex A1/A2 (3.97 kcal/mol), the introduction of s^2^U at the 5′ end of the strand (A3) should not change the overall thermodynamic asymmetry of the siRNA. Accordingly, strand selection should not be changed. However, it can be expected that the efficiency of strand selection may be decreased *i*.*e*. the lower gene silencing potency of A4 in reference to A3 would be observed, since the difference in the stability of siRNA 5′ ends is reduced. Cell line experiments show that the introduction of this modified nucleotide has a major impact on the choice of antisense strand by the RISC complex (Fig. 7A). The use of A3 strand results in decrease of the ZsGreen1 mRNA level by 21.2% Thus, silencing by the A4 strand is greater than by A2 and results in the mRNA expression reduction by 90.6% relative to the control. Introduction of s^2^U causes a large difference in the use of both tested strands as an antisense strand. Considering the A4 strand as the desired guide strand, the chance for the appearance of sense pathway off-target effects has been reduced. Since s^2^U does not change 5′ end thermodynamic asymmetry of A3/A4 siRNA and based on thermodynamic theory about strand selection, the A3 should be selected as the antisense strand. The observed change in strand selection in this instance might be due to low RISC tolerance for the presence of s^2^U in the guide strand. In the B1/B2 duplex, in order to reduce thermodynamic stability at 5′ end of the B1 strand, 2-thiocytidine (s^2^C) was introduced into penultimate position (B3/B4). The UV melting experiments show changes in free energy after the introduction of this modified nucleotide by 1.32 kcal/mol (Table S1, supplementary file). Despite the addition of this modified nucleotide, the end with the G-C pair is more stable and, according to the less stable 5′-end theory, the B4 strand should still be preferentially chosen as antisense strand. The presence of this modification within the siRNA strongly blocks the biological activity of the B3/B4 duplex (Fig. 7B). The B3 strand causes a reduction in the expression of the ZsGreen1 gene mRNA of only 22.8% and the B4 strand of 37.4%. Presumably, the presence of s^2^C within siRNA is detrimental for RISC activity.

**Figure 8:**
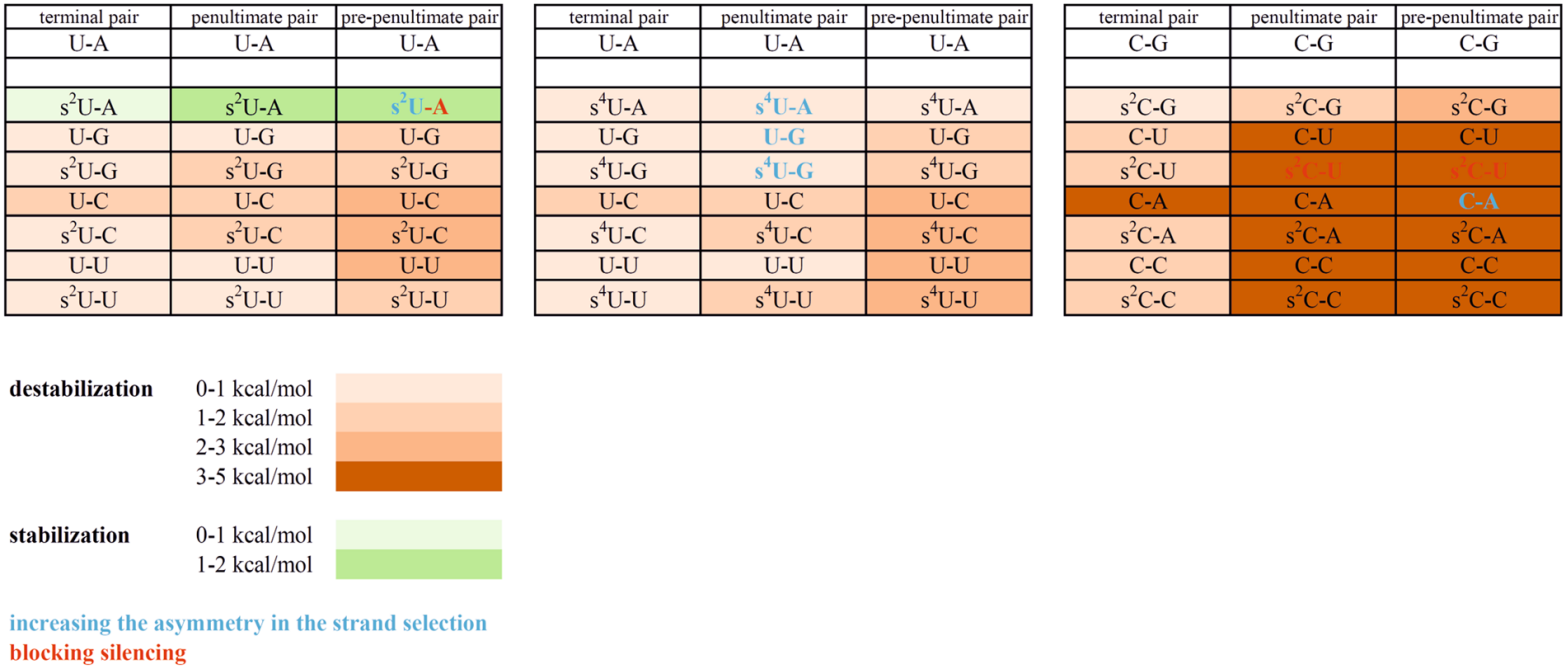
Schematic representation of the effect of mismatches and modified nucleotides on the thermodynamic stability of siRNA model duplexes in terminal, penultimate and pre-penultimate position. In addition, the pairs that were introduced to the full-length siRNA and used in HeLa cell line experiments were marked and the impact of the strand selection by RISC complex were noted.

For duplexes, which have the same defined thermodynamic stability of the ends, it was decided to introduce more changes, since it is easier to investigate the effect of thermodynamic asymmetry for this type of siRNAs. To the duplex C1/C2, changes were introduced to destabilize one end of the duplex, mismatch C-A was introduced at the two ends of the duplex so the strand with a mismatch at the 5′-end should be selected as antisense strand (C3 and C8 strand); introduction of s^2^C should also result in preferential selection of C6 as antisense strand. Duplex C3/C4 possesses C-A mismatch in pre-penultimate position (Fig. 7C). UV melting experiments show that this change causes a decrease in thermodynamic stability by 4.39 kcal/mol (Table S1, Supplementary File). The studies on HeLa cell lines indicate that this change causes a large difference in the use of both strands as guide strands. The C3 strand knocks-down mRNA expression of the ZsGreen1 gene by 69.6%. In contrast, the use of an C3/C4 duplex and a plasmid with the complementary region to the C4 strand results in increased expression of the ZsGrenn1 gene mRNA to the level of 200.9% in relation to the control. In the presence of s^2^C at pre-penultimate position of C5/C6 (Fig. 7D), the activity of the siRNA molecule is completely blocked and the promotion of mRNA expression of the ZsGreen1 gene is observed to the level of 175.4% and 556.7% relative to the control. This observation, as well as our previous analysis of B3/B4 ability to knock-down ZsGreen1, confirms that s^2^C seems to be incompatible with the siRNA pathway. Analogously to the duplex C3/C4, the C7/C8 duplex also has C-A mismatch at one end (Fig. 7E). To check whether the trend in changing the strand selection is similar, this duplex slightly differs from the C3/C4 duplex in the neighborhood of the C-A mismatch. Quantitative results of qPCR show that in this instance a large discrepancy is observed in the use of strands as guide strand by RISC (Fig. 7E). The C7 strand increases the expression of the ZsGreen1 gene mRNA in relation to the control to the level of 198.9% and the C8 strand reduces the expression by 90.7%. Chemical modifications or mismatches were also introduced into the D1/D2 duplex, resulting in the formation of D3/D4, D5/D6, D7/D8, and D9/D10 siRNA duplexes. It was expected that s^2^U would increase the thermodynamic stability, while the other changes were supposed to destabilize one end. According to the less stable 5′-end theory, it was expected that the D3, D5, D7, and D9 strands would be selected as the antisense strands. The presence of the 2-thiouridine (s^2^U) within the pre-penultimate position of the D4 strand (Fig. 7F) resulted in an increase in the siRNA thermodynamic stability by 1.38 kcal/mol (Table S2, Supplementary Information). It was expected that the 5′-end of D4 would have higher thermodynamic stability and thereby lower activity as an antisense strand. The results from qPCR show that the activity of this siRNA is low. The reduction in the expression of the ZsGreen1 mRNA in the case of the D3 strand is 28.7%, while for the D4 strand it is only 13.4%. As for the A3/A4 duplex, the strand with an s^2^U modified nucleotide is not able to efficiently knock-down gene expression. Nevertheless, in case of the D3/D4 duplex the action of the second strand also results in poor degradation of the ZsGreen1 mRNA. In order to trigger siRNA strand bias, we also decreased the thermodynamic stability of the siRNA end by the introduction of G-U mismatch at penultimate position (D5/D6 duplex). According to the UV melting experiments, this modified nucleotide causes duplex destabilization by 0.81 kcal/mol. As in the previous examples, the introduction of the mismatch results in a large diversification of the use of the strands in this duplex by the human RISC complex. The D5 strand decreases mRNA expression of the ZsGreen1 gene to 17.4%, while the second strand D6 increases expression to 165% (Fig. 7G). Another way to decrease thermodynamic stability of one of the siRNA ends is the introduction of s^4^U-G mismatch (duplex D7/D8, penultimate position) (Fig. 7H). According to the UV melting experiments, such a configuration causes a decrease in the thermodynamic stability of the model siRNA duplex by 0.54 kcal/mol (Table S3, Supplementary Information). However, the results of cell line experiments show that the strand which has a more stable 5′-end and contains s^4^U (D8) is more preferably chosen by RISC. The effect of D7 action is promotion of ZsGreen1 mRNA expression up to 134.7%, whereas the D8 strand causes a 67.1% reduction of gene expression. The last duplex tested on the HeLa cell line was the D9/D10 containing s^4^U which was involved in the formation of the canonical base pair s^4^U-A (Fig. 7I). The single s^4^U residue at penultimate position of the helix reduces the thermodynamic stability of siRNA by 0.26 kcal/mol (Table S3, Supplementary Information). However, compared to a duplex without modification (D1/D2), there is a substantial difference in the selection of antisense strand by the RISC. The strand with the less stable 5′-end (D1) does not significantly reduce the expression of ZsGreen1 gene mRNA (ZsGreen1 level is 84.3% relative to the control). In contrast, the strand containing s^4^U diminishes this level by 99.5%. The two presented examples of siRNA duplexes with s^4^U substitution may suggest that the Ago2 protein preferentially selects the strand with this modified nucleotide as an antisense strand. Summing up the results of cellular research, the changes caused by the insertion of mismatches or modified nucleotides into the siRNA molecule can be divided into these that increase the asymmetry of the strand selection (and thus reduce the off-target effects) and these that block the silencing of the reporter gene (Fig. 8).

It is also worth noting the case where the expression of the ZsGreen1 mRNA was increased compared to the control. It was observed for strands C4, C5, C6, C7, D6, D7 used as an antisense strand. In 2006 it was found that small double-stranded RNA molecules targeted towards promoter may increase the gene expression^44^. This process is called RNA activation (RNAa) and molecules involved in this process are small activating RNAs (saRNAs). The mechanism of this process is not fully understood. The molecules that activate this process are known to be siRNA mimics (19 nucleotide duplex and 3′-dangling ends of dTdT)^44^. In mammalian cells, the RNAa mechanism requires the Ago2 protein^45,46^ just like RNAi. Nevertheless, there are substantial differences in the kinetics of these reactions. The RNAi mechanism is activated within a few hours after siRNA has been added to the cell and takes about 5–7 days^47,48^. In contrast, the enhancement of gene expression by the saRNA molecule occurs after 24–48 hours and the molecule can be detected after approximately 2 weeks^44,47^. As previously mentioned, this process is not thoroughly examined. However, it is possible that the introduction of mismatches or modified nucleotides into siRNA molecules can activate or enhance the RNAa mechanism and increase the expression of the reporter gene relative to the control.

The introduction of modified nucleotides and mismatches confirms earlier observations that in the human HeLa cell line the RISC does not select the guide strand solely based on the difference in siRNA 5′-end stability. Only the introduction of mismatches at specified positions causes the situation where antisense strand selection is based on siRNA strand bias and lower stability of one of the 5′-ends. Earlier reports also show that the theory of a less stable 5′-end is not universal^49^, but when the mismatches are introduced into siRNA this hypothesis seems to work well^20^. On the other hand, the introduction of modified nucleotides, where stabilizing or destabilizing properties have been demonstrated, does not confirm this theory. Based on the research presented herein, it can be concluded that some modified nucleotides are not tolerated by RISC (s^2^U, s^2^C), whereas some are even desirable (s^4^U).

## Conclusions

Efficient and effective therapy based on siRNA requires the well-known characteristics of siRNA duplexes and precise knowledge of the influence of particular modifications is pivotal to change siRNA action in a controlled manner. After the discovery of the mechanisms of RNA interference, there has been great interest by pharmaceutical companies in siRNA molecules. Subsequently, some problems have emerged connected the use of these molecules. The main problems are: cell delivery, off-target effects, immune response, and serum stability of the molecule. There are many examples of the introduction of modified nucleotides to increase the stability of the siRNA molecules in serum, including conjugation of nanoparticles with siRNA to facilitate cell membrane passage^15,50^. Herein we have presented an comprehensive analysis of the use of individual strands within the siRNA duplex as guide strands depending on the thermodynamic stability of the ends. The results confirm that for the HeLa cell line, each of the siRNA strands has the potential to become the antisense strand. Therefore, this situation is highly unfavorable when we want to use siRNA as a therapeutic agent. Even if a strand that is considered the antisense one causes a large reduction in the expression of a specified gene, a second strand can be also introduced into the active RISC. As a consequence, undesired off-target effects could be observed. In addition, the lack of siRNA specificity of action may increase the immune response and mean that a higher siRNA concentration will be needed for the best activity of the siRNA molecule. RISC proteins can be unproductively activated, which, when combined with the information that the same proteins in humans participate in the miRNA mechanism, results in a high concentration of siRNA that can alter the activity of genes controlled by miRNA. The presented changes in thermodynamic stability by introducing mismatches result in the use of only one strand as antisense according to the theory of the less stable 5′-end. When modified nucleotides were introduced to change the stability of the ends, the effect on the antisense strand selection was unpredictable. Some modifications are unacceptable (s^2^U, s^2^C), which others are even desirable (s^4^U). The presented data clearly showed that a few changes can lead to a situation, where only one strand is selected as an antisense strand, which in the case of using siRNAs as a therapeutic agent seems to be crucial.

## Materials and Methods

### Oligonucleotides synthesis

The oligonucleotides were synthesized using MerMade12 (BioAutomation) synthesizers and β-cyanoethyl phosphoramidite chemistry^51^. Commercially available A, C, G, U, s^2^U, s^4^U, s^2^C phosphoramidites were used to synthesize modified and unmodified oligonucleotides (ChemGenes, Gene-Pharma). The details of oligoribonucleotide deprotection and purification were previously described^24^. Deprotection of oligonucleotides modified with s^2^U, s^4^U, and s^2^C was performed by the treatment with methanol/aqueous ammonia solution (1:1 v/v) at room temperature for 48 h. Thin-layer chromatography (TLC) purification of the model siRNA oligonucleotides was performed for model siRNA strands using Merck 60 F254 TLC plates with the mixture 1-propanol/aqueous ammonia/water D 55/35/10 (v/v/v). Full length siRNAs strands were purified via 12% polyacrylamide gel electrophoresis in denaturing conditions. The composition of all oligonucleotides was confirmed by MALDI-TOF mass spectrometry (Table S5, Supplementary Information).

### Plasmids and siRNAs

The sequences of full length siRNA strands used in this study are as follows: A1/A2 (A1 5′ UAUGCAAGGCUAGCCCGCGTT, A2 5′ CGCGGGCUAGCCUUGCAUATT), B1/B2 (B1 5′ GCGGCAAGGCUAGCCCAUATT, B2 5′ UAUGGGCUAGCCUUGCCGCTT), C1/C2 (C1 5′ CGCGCAAGGCUAGCCCGCGTT, C2 5′ CGCGGGCUAGCCUUGCGCGTT), D1/D2 (D1 5′ UAUGCAAGGCUAGCCCAUATT, D2 5′ UAUGGGCUAGCCUUGCAUATT), A3/A4 (A3 5′ UAs^2^UGCAAGGCUAGCCCGCGTT, A4 5′ CGCGGGCUAGCCUUGCAUATT), B3,/B4 (B3 5′ Gs^2^CGGCAAGGCUAGCCCAUATT, B4 5′ UAUGGGCUAGCCUUGCCGCTT), C3/C4 (C3 5′ CGCGCAAGGCUAGCCCGCGTT, C4 5′ CGCGGGCUAGCCUUGCACGTT), C5/C6 (C5 5′ CGCGCAAGGCUAGCCCGCGTT, C6 5′ CGs^2^CGGGCUAGCCUUGCGCGTT), C7/C8 (C7 5′ CGCGCAAGGCUAGCCCACGTT, C8 5′ CGCGGGCUAGCCUUGCGCGTT), D3/D4 (D3 5′ UAUGCAAGGCUAGCCCAUATT, D4 5′ UAs^2^UGGGCUAGCCUUGCAUATT), D5/D6 (D5 5′ UGUGCAAGGCUAGCCCAUATT, D6 5′ UAUGGGCUAGCCUUGCAUATT), D7/D8 (D7 5′ UGUGCAAGGCUAGCCCAUATT, D8 5′ UAUGGGCUAGCCUUGCAs^4^UATT), D9/D10 (D9 5′ UAUGCAAGGCUAGCCCAUATT, D10 5′ UAUGGGCUAGCCUUGCAs^4^UATT) and self-complementary control siRNA (5′ UCAUACUUGCAAGUAUGATT).

pZsGreen1-N1 is a human codon-optimized expression vector that encodes a variant of the *Zoanthus sp*. green fluorent protein ZsGreen1. Plasmid pZsGreen1-N1 allows cloned genes to be inserted into the multiple cloning site upstream of the ZsGreen1 coding sequences. For ZsGreen expression plasmid construct, sequences complementary to each siRNA were cloned. The plasmid and proper oligonucleotide were cut with restriction enzymes BamHI and HindIII. The following plasmids were designed:

P1 (insert: CGATCAAGCTTAGCCAACGCGGGCTAGCCTTGCATAATGCGGATCCAAGCC), P2 (insert: CGATCAAGCTTAGCCAATATGCAAGGCTAGCCCGCGATGCGGATCCAAGCC), P3 (insert: CGATCAAGCTTAGCCAATATGGGCTAGGCTTGCCGCATGCGGATCCAAGCC), P4 (insert: CGATCAAGCTTAGCCAAGCGGCAAGGCTAGCCCATAATGCGGATCCAAGCC), P5 (insert: CGATCAAGCTTAGCCAACGCGGGCTAGGCTTGCGCGATGCGGATCCAAGCC), P6 (insert: CGATCAAGCTTAGCCAACGCGCAAGGCTAGCCCGCGATGCGGATCCAAGCC), P7 (insert: CGATCAAGCTTAGCCAATATGGGCTAGGCTTGCATAATGCGGATCCAAGCC), P8 (insert: CGATCAAGCTTAGCCAATATGCAAGGCTAGCCCATAATGCGGATCCAAGCC).

### HeLa culture and transfection

HeLa cells were cultivated as described earlier^52^. The passage of 100.000 cells in each of the 24 wells was performed. After 24 h, transfection was performed using Lipofectamine 2000 in accordance with the manufacturer’s recommendations. To evaluate strand selection of siRNA by RISC complex, HeLa cells were cotransfected with the 1 µg of suitable plasmid (P1–P8) and 10 nM concentration of siRNA molecules (A1/A2, B1/B2, C1/C2, D1/D2, A3/A4, B3/B4, C3/C4, C5/C6, C7/C8, D3/D4, D5/D6, D7/D8, D9/D10). The transfected cells were grown for 24 h at 37 °C in a 5% CO_2_ atmosphere without antibiotics in medium. As a control siRNA with random sequence was used. The RNA from the cultured cells using acid guanidinium thiocyanate-phenol-chloroform extraction was isolated, and the RNA was treated with DNase I. The quality of the isolated RNA was verified by agarose gel electrophoresis.

### MTT assay

The half maximal inhibitory concentration (IC_50_) of siRNAs was determined by MTT assay. The HeLa cells were passaged in 96-well plates in the amount of 5000 cells per well. The cells were incubated for 24 hours at 37 °C in a 5% CO_2_ atmosphere in the in RPMI-1640 medium supplemented with 10% FBS, vitamins and antibiotics. After incubation the cells were transfected with different concentrations of siRNAs (20, 50, 100, 200, 500 nM) using Lipofectamine 2000. After 24 h incubation the MTT assay was performed. The medium was discarded from the cells, then 150 µl of MTT solution (0.5 mg/ml) in medium without phenol red was added. The MTT reagent was incubated with the cells for 2 hours, next MTT solution was discarded and water-insoluble formazan crystals were dissolved in 40 mM HCl in isopropanol. The absorbance was measured at 550 nm and 650 nm as reference on XMark Microplate Spectrophotometer. The IC_50_ value was estimated using dose-response curves.

### RT-qPCR analysis

Earlier prepared 500 ng RNA template was used for cDNA synthesis, using the iScript cDNA Synthesis Kit (Bio-Rad). The qPCR was performed on a CFX96 real-time PCR system (Bio-Rad) using iTaq SYBR Green Supermix (Bio-rad) and 96-well clear plates. The level of ZsGreen1 mRNA was quantified with the use of target gene primers: J9 5′-GTACCACGAGTCCAAGTTCTAC, J10 5′-CCAGTTGTCGGTCATCTTCTT, and normalized to human β-actin levels, reference gene primers: J5 5′-GCCAGCAGCCTCTGATCTG, J6 5′-CTGGTTCTTGCCAGCCTCTAG. The Ct values of human β-actin was in the range of 18–23. The qPCR cycles were as follows: 95 °C for 5 min; (95 °C for 10 sec and 66 °C 20 sec) for 40 cycles.

### qPCR statistical analysis

Statistical analysis of the qPCR results was performed in analogy to the described earlier method^53^. PCR efficiency of each reaction was ranged from 92% to 99%. The results from the biological replicates for particular samples were gathered to determine the mean normalized expression and its standard deviation (Bio-Rad CFX Manager 3.0). The normalized relative expression of ZsGreen1 from biological replicates for siRNAs and control siRNA was compared at a significance level of 0.05 or 0.01 using Bio-rad CFX Manager 3.0. Statistically significant differences in mean expression between tested siRNAs and control siRNA (P < 0.05 or P < 0.01) were observed but not in all samples.

### UV melting experiments

The siRNA duplexes were melted in buffer containing 100 mM NaCl, 20 mM sodium cacodylate, 0.5 mM Na_2_EDTA, pH 7. The single strand oligonucleotide concentrations were calculated based on absorbance at 80 °C, and the single strand extinction coefficients were calculated on the website www.ribotask.com. The measurements were performed for 9 different concentrations of each model siRNA in the range 10^−4^–10^−6^ M. Absorbance vs. temperature melting curves were measured at 260 nm at the heating rate 1 °C/min from 0 to 90 °C using a Beckman DU 640 or JASCO V-650 spectrophotometer with a thermoprogrammer. The melting curves were analyzed, and the thermodynamic parameters were calculated using a two-state model with the MeltWin 3.5 software^54^. For all sequences, the ΔH° values derived from the T_M_^−1^ vs. ln(CT/4) plots were within 15% of the ΔH° values derived from averaging the fits to the individual melting curves, indicating that the two-state model is reasonable.

## Electronic supplementary material

Supplementary material
