# Supplementary material for: A systematic study on the influence of thermodynamic asymmetry of 5′-ends of siRNA duplexes in relation to their silencing potency
Source: Sci Rep. 2019 Feb 21;9:2477. doi: 10.1038/s41598-018-36620-9 (PMC6385221; doi:10.1038/s41598-018-36620-9)
Supplement: Supplementary file 1 — Supplementary material [file 41598_2018_36620_MOESM1_ESM.pdf]

SUPPLEMENTARY MATERIAL

A systematic study on the influence of thermodynamic asymmetry of 5'-ends of siRNA duplexes in relation to their silencing potency

*Jolanta Lisowiec-Wąchnicka, Natalia Bartyś and Anna Pasternak**

[1]Department of Nucleic Acids Bioengineering, Institute of Bioorganic Chemistry, Polish Academy of Sciences, Noskowskiego 12/14, 61-704 Poznan, Poland

**Correspondence should be addressed to A.P. Tel:* + 48 618 528 503*; Fax: +48 618 520 532; Email*: apa@ibch.poznan.pl

Table S1. Thermodynamic parameters of model siRNA helix formation modified with 2-thiocytidine (s2C)[a].

Duplexes (5'-3')	Average of curve fits				T_M^{-1} vs log C_T plots							
	**-ΔH° (kcal/mol)**	**-ΔS° (eu)**	**-ΔG°$_{37}$ (kcal/mol)**	**T_M ^{b} (°C)**	**-ΔH° (kcal/mol)**	**-ΔS° (eu)**	**-ΔG°$_{37}$ (kcal/mol)**	**T_M^b (°C)**	**ΔΔG°$_{37}$ (kcal/mol)***	**ΔT_M^b (°C)***	**ΔΔG°$_{37}$ (kcal/mol) ***	**ΔT_M^b (°C)***
CUGUGCACAGTT	94.6±0.9	260.4±2.7	13.88±0.08	66.4	92.4±1.3	253.6±3.8	13.71±0.09	66.5	0	0		
s2CUGUGCACAGTT	94.7±3.2	264.3±9.9	12.74±0.15	62.0	97.6±4.7	273.1±14.3	12.91±0.30	61.8	0.40	-2.4	0	0
CUGUGCACAUTT	76.2±4.1	212.7±12.3	10.23±0.25	56.7	75.3±5.3	209.8±16.3	10.18±0.27	56.8	1.77	-4.9		
s2CUGUGCACAUTT	71.4±3.3	197.5±10.1	10.11±0.20	57.5	78.0±3.2	217.9±9.7	10.42±0.15	57.1	1.65	-4.7	1.25	-2.4
CUGUGCACAATT	47.8±8	141.6±22.8	3.86±0.36	25.6	47.1±4.2	139.1±14.2	3.92±0.28	25.9	4.90	-20.3		
s2CUGUGCACAATT	79.2±5.3	221.3±16.2	10.59±0.31	57.5	73.8±4.0	204.7±12.3	10.34±0.19	57.9	1.69	-4.3	1.29	-2.0
CUGUGCACACTT	74.5±5.5	206.1±16.9	10.61±0.32	59.0	64.2±4.2	174.7±12.8	10.04±0.26	59.6	1.84	-3.5		
s2CUGUGCACACTT	79.0±5.7	221.0±17.5	10.48±0.28	57.1	73.2±3.7	203.1±11.3	10.19±0.18	57.4	1.76	-4.6	1.36	-2.2
ACUUGCAAGUTT	91.2±1.6	258.0±5.0	11.15±0.11	56.8	89.9±2.3	254.2±7.1	11.08±0.11	56.8	0	0		
As2CUUGCAAGUTT	77.0±6.3	221.4±19.7	8.31±0.20	48.0	86.1±5.3	250.4±16.9	8.44±0.11	47.3	1.32	-4.8	0	0
ACUUGCAAUUTT	56.2±6.8	166.8±22.2	4.45±0.22	30.4	56.3±3.3	167.1±11.2	4.46±0.15	30.5	3.31	-13.2		
As2CUUGCAAUUTT	54.5±6.0	161.4±19.9	4.49±0.16	30.4	53.5±3.9	157.8±13.1	4.57±0.16	30.7	3.26	-13.1	1.94	-8.3
ACUUGCAAAUTT	49.3±4.6	144.3±15.4	4.53±0.19	30.0	51.4±2.9	151.5±9.6	4.45±0.14	29.7	3.32	-13.6		
As2CUUGCAAAUTT	51.8±6.9	152.3±22.4	4.53±0.11	30.3	56.5±5.7	168.2±19.4	4.32±0.33	29.7	3.38	-13.6	2.06	-8.8
ACUUGCAACUTT	49.7±2.7	146.0±9.2	4.42±0.13	29.3	48.5±2.4	141.9±8.0	4.46±0.13	29.4	3.31	-13.7		

As2CUUGCAACUTT	50.4±3.3	148.6±11.0	4.34±0.10	29.0	50.6±2.2	149.1±7.4	4.33±0.11	28.9	3.38	-14.0	2.01	-9.2
UACUGCAGUATT	90.9±0.9	253.8±2.5	12.19±0.15	60.9	87.3±4.1	242.7±12.3	11.98±0.24	61.1	0	0		
UAs2CUGCAGUATT	63.7±1.7	180.7±5.3	7.63±0.05	46.8	64.1±0.5	181.9±1.5	7.64±0.01	46.8	2.17	-7.2	0	0
UACUGCAUUATT	62.8±5.0	194.7±17.0	2.42±0.26	21.7	65.7±5.2	204.7±17.8	2.24±0.37	21.6	4.87	-19.8		
UAs2CUGCAUUATT	55.1±5.9	168.5±19.8	2.79±0.22	21.5	59.5±5.9	184.0±20.2	2.46±0.43	21.1	4.76	-20.0	2.59	-12.9
UACUGCAAUATT	56.9±5.3	174.5±17.9	2.75±0.32	21.8	50.0±5.2	150.8±18.0	3.21±0.40	22.4	4.39	-19.4		
UAs2CUGCAAUATT	47.6±2.1	142.7±7.0	3.31±0.16	22.3	52.0±4.8	157.9±16.7	2.99±0.35	21.8	4.50	-19.7	2.33	-12.5
UACUGCACUATT	53.1±1.3	162.5±4.3	2.74±0.10	20.7	56.3±3.3	173.6±11.4	2.46±0.28	20.3	4.76	20.4		
UAs2CUGCACUATT	62.1±0.7	191.4±2.3	2.70±0.03	22.8	69.2±5.4	215.9±18.5	2.21±0.38	22.2	4.89	-19.5	2.72	-12.3

a – solutions: 100 mM NaCl, 20mM sodium cacodylate, 0.5 mM Na_2EDTA, pH 7, b - calculated for 10^{-4} M oligomer concentration; *per single modification/mismatch

Table S2. Thermodynamic parameters of model siRNA helix formation modified with 2-thiouridine (s2U)[a].

Duplexes (5'-3')	Average of curve fits				T_M^{-1} vs log C_T plots							
	**-ΔH˚ (kcal/mol)**	**-ΔS˚ (eu)**	**-ΔG˚37 (kcal/mol)**	**T_M b (˚C)**	**-ΔH˚ (kcal/mol)**	**-ΔS˚ (eu)**	**-ΔG˚37 (kcal/mol)**	**T_M^b (˚C)**	**ΔΔG˚37 (kcal/mol)***	**ΔT_M^b (˚C)***	**ΔΔG˚37 (kcal/mol) ***	**ΔT_M^b (˚C)***
UUGUGCACAATT	84.2±2.2	233.9±6.9	11.68±0.09	60.8	89.6±1.4	250.1±4.2	11.97±0.08	60.5	0	0		
s2UUGUGCACAATT	87.3±7.0	241.2±21.4	12.53±0.35	63.4	84.4±9.7	232.1±29.4	12.38±0.65	63.8	-0.21	1.7	0	0
UUGUGCACAGTT	79.0±3.1	220.1±9.7	10.69±0.12	58.0	83.4±3.8	233.8±11.8	10.89±0.19	57.7	0.54	-1.4		
s^{2}UUGUGCACAGTT	75.9±5.0	210.6±15.5	10.60±0.26	58.5	78.5±2.6	218.6±8.1	10.70±0.13	58.2	0.64	-1.2	0.84	-2.8
UUGUGCACACTT	71.9±4.6	199.5±14.2	10.01±0.24	56.9	70.5±2.2	195.4±6.8	9.92±0.10	56.8	1.03	-1.9		
s^{2}UUGUGCACACTT	85.6±7.3	241.5±22.6	10.68±0.41	56.3	78.2±5.9	218.7±18.0	10.34±0.30	56.7	0.82	-1.9	1.02	-3.6
UUGUGCACAUTT	76.8±5.6	213.3±16.8	10.70±0.37	58.7	79.1±1.7	220.0±5.2	10.86±0.08	58.7	0.56	-0.9		
s2UUGUGCACAUTT	82.3±1.2	227.8±3.5	11.68±0.12	61.4	78.2±2.4	215.3±7.3	11.44±0.14	61.7	0.27	0.6	0.47	-1.8
AUUUGCAAAUTT	75.2±2.4	221.8±7.9	6.43±0.05	40.2	76.9±1.2	227.2±3.8	6.43±0.01	40.1	0	0		
As2UUUGCAAAUTT	87.4±6.0	256.0±19.4	9.25±0.24	50.2	81.7±13.8	234.0±	9.08±0.66	50.5	-1.33	5.2	0	0
AUUUGCAAGUTT	53.5±3.6	156.7±11.5	4.90±0.15	32.5	56.8±4.9	167.5±16.2	4.81±0.17	32.4	0.82	-3.9		
As2UUUGCAAGUTT	65.0±7.5	194.7±25.0	4.66±0.23	32.2	71.0±4.2	214.7±13.8	4.42±0.15	31.6	1.01	-4.3	2.33	-9.45
AUUUGCAACUTT	44.0±1.5	128.1±4.9	4.22±0.09	27.1	42.5±1.4	123.1±4.8	4.31±0.08	27.4	1.06	-6.4		
As^{2}UUUGCAACUTT	39.3±5.7	111.9±19.1	4.62±0.32	28.9	42.7±4.7	123.6±16.0	4.36±0.33	27.7	1.04	-6.2	2.36	-11.4
AUUUGCAAUUTT	59.0±0.9	174.0±3.1	5.03±0.09	33.7	64.2±1.8	191.3±5.8	4.83±0.07	33.0	0.8	-3.6		

As2UUUGCAAUUTT	62.7±7.1	182.2±23.1	6.21±0.28	39.6	65.2±9.1	190.4±29.3	6.16±0.30	39.3	0.14	-0.4	1.51	-5.6
UAUUGCAAUATT	76.4±1.9	220.4±6.4	8.02±0.13	46.8	78.5±5.7	227.1±18.1	8.06±0.12	46.7	0	0		
UAs2UUGCAAUATT	89.0±4.6	251.6±14.6	10.96±0.11	56.6	85.5±9.2	240.8±28.4	10.82±0.45	56.9	-1.38	5.1	0	0
UAUUGCAGUATT	80.8±3.7	243.3±11.9	5.32±0.10	35.6	83.6±4.8	252.7±15.6	5.26±0.10	35.4	1.40	-5.7		
UAs^{2}UUGCAGUATT	43.6±6.6	122.0±21.2	5.78±0.16	37.8	39.0±5.3	107.2±17.3	5.80±0.27	38.0	1.13	-4.4	2.51	-9.5
UAUUGCACUATT	53.0±2.4	163.4±7.8	2.30±0.19	18.4	46.3±6.5	140.3±22.6	2.81±0.55	18.9	2.63	-13.9		
UAs^{2}UUGCACUATT	50.3±4.6	152.3±15.6	3.06±0.31	21.6	58.5±5.3	180.6±18.3	2.46±0.39	20.8	2.80	-13.0	4.18	-18.1
UAUUGCAUUATT	49.1±4.4	147.3±15.1	3.35±0.33	23.0	47.8±1.7	143.0±5.8	3.41±0.11	22.9	2.33	-11.9		
UAs2UUGCAUUATT	68.4±4.9	200.7±16.0	6.12±0.10	39.0	73.8±4.8	218.4±15.7	6.04±0.10	38.5	1.01	-4.1	2.39	-9.2

a – solutions: 100 mM NaCl, 20mM sodium cacodylate, 0.5 mM Na_2EDTA, pH 7, b - calculated for 10^{-4} M oligomer concentration; *per single modification/mismatch

Table S3. Thermodynamic parameters of model siRNA helix formation modified with 4-thiouridine (s4U)[a].

Duplexes (5'-3')	Average of curve fits				T_M^{-1} vs log C_T plots							
	**-ΔH° (kcal/mol)**	**-ΔS° (eu)**	**-ΔG°$_{37}$ (kcal/mol)**	**T_M [b] (°C)**	**-ΔH° (kcal/mol)**	**-ΔS° (eu)**	**-ΔG°$_{37}$ (kcal/mol)**	**T_M[b] (°C)**	**ΔΔG°$_{37}$ (kcal/mol)***	**ΔT_M[b] (°C)***	**ΔΔG°$_{37}$ (kcal/mol) ***	**ΔT_M[b] (°C)***
UUGUGCACAATT	84.2±2.2	233.9±6.9	11.68±0.09	60.8	89.6±1.4	250.1±4.2	11.97±0.08	60.5	0	0		
s4UUGUGCACAATT	83.3±0.6	232.0±2.0	11.34±0.08	59.6	78.9±4.3	218.6±13.1	11.12±0.22	60.0	0.43	-0.25	0	0
UUGUGCACAGTT	79.0±3.1	220.1±9.7	10.69±0.12	58.0	83.4±3.8	233.8±11.8	10.89±0.19	57.7	0.54	-1.4		
s4UUGUGCACAGTT	74.3±6.9	205.2±21.1	10.69±0.36	59.4	82.8±2.5	231.1±7.7	11.09±0.14	58.7	0.44	-0.9	0.02	-0.7
UUGUGCACACTT	71.9±4.6	199.5±14.2	10.01±0.24	56.9	70.5±2.2	195.4±6.8	9.92±0.10	56.8	1.03	-1.9		
s4UUGUGCACACTT	74.5±4.6	207.7±14.0	10.08±0.27	56.5	72.4±5.5	201.3	9.99±0.24	56.6	0.99	-2.0	0.57	-1.7
UUGUGCACAUTT	76.8±5.6	213.3±16.8	10.70±0.37	58.7	79.1±1.7	220.0±5.2	10.86±0.08	58.7	0.56	-0.9		
s4UUGUGCACAUTT	75.4±3.2	210.5±9.7	10.12±0.21	56.4	80.8±2.1	227.2±6.6	10.36±0.09	56.1	0.81	-2.2	0.38	-2.0
AUUUGCAAAUTT	75.2±2.4	221.8±7.9	6.43±0.05	40.2	76.9±1.2	227.2±3.8	6.43±0.01	40.1	0	0		
As4UUUGCAAAUTT	67.5±7.1	198.4±23.0	5.94±0.11	38.2	64.9±2.4	190.3±7.8	5.91±0.04	38.1	0.26	-1.0	0	0
AUUUGCAAGUTT	53.5±3.6	156.7±11.5	4.90±0.15	32.5	56.8±4.9	167.5±16.2	4.81±0.17	32.4	0.82	-3.9		
As4UUUGCAAGUTT	28.3±7.3	73.4±24.3	5.50±0.30	35.1	32.2±2.1	86.5±7.0	5.35±0.09	33.9	0.54	-3.1	0.28	-2.1
AUUUGCAACUTT	44.0±1.5	128.1±4.9	4.22±0.09	27.1	42.5±1.4	123.1±4.8	4.31±0.08	27.4	1.06	-6.4		
As4UUUGCAACUTT	46.5±5.5	137.0±18.1	3.96±0.14	26.0	53.5±5.9	161.1±20.0	3.56±0.38	25.2	1.44	-7.5	1.18	-6.5
AUUUGCAAUUTT	59.0±0.9	174.0±3.1	5.03±0.09	33.7	64.2±1.8	191.3±5.8	4.83±0.07	33.0	0.8	-3.6		

As4UUUGCAAUUTT	50.6±6.2	148.8±21.0	4.39±0.30	29.3	53.5±3.0	158.9±10.2	4.23±0.14	28.9	1.10	-5.6	0.84	-4.6
UAUUGCAAUATT	76.4±1.9	220.4±6.4	8.02±0.13	46.8	78.5±5.7	227.1±18.1	8.06±0.12	46.7	0	0		
UAs4UUGCAAUATT	59.1±4.9	168.7±15.9	6.79±0.11	42.9	61.6±3.9	176.9±12.7	6.76±0.07	42.5	0.65	-2.1	0	0
UAUUGCAGUATT	80.8±3.7	243.3±11.9	5.32±0.10	35.6	83.6±4.8	252.7±15.6	5.26±0.10	35.4	1.40	-5.7		
UAs4UUGCAGUATT	69.8±15.9	210.0±52.2	4.64±0.33	32.4	70.6±7.4	213.2±24.5	4.49±0.29	31.9	1.79	-7.4	1.14	-5.3
UAUUGCACUATT	53.0±2.4	163.4±7.8	2.30±0.19	18.4	46.3±6.5	140.3±22.6	2.81±0.55	18.9	2.63	-13.9		
UAs4UUGCACUATT	47.4±3.4	142.8±11.8	3.09±0.29	21.0	51.1±4.0	155.8±14.0	2.78±0.31	20.4	2.64	-13.2	1.99	-11.1
UAUUGCAUUATT	49.1±4.4	147.3±15.1	3.35±0.33	23.0	47.8±1.7	143.0±5.8	3.41±0.11	22.9	2.33	-11.9		
UAs4UUGCAUUATT	52.1±4.7	158.6±15.7	2.94±0.22	21.5	52.7±6.7	160.8±23.2	2.87±0.50	21.3	2.60	-12.7	1.95	-10.6

a – solutions: 100 mM NaCl, 20mM sodium cacodylate, 0.5 mM Na_2EDTA, pH 7, b - calculated for 10^{-4} M oligomer concentration; *per single modification/mismatch

Table S4

half maximal inhibitory concentration IC_{50} [µM] in HeLa cell line after 24h incubation with unmodiefied siRNAs			
A1/A2	B1/B2	C1/C2	D1/D2
1.44	1.22	2.81	2.45

half maximal inhibitory concentration IC_{50} [µM] in HeLa cell line after 24h incubation with modiefied siRNAs								
A3/A4	B3/B4	C3/C4	C5/C6	C7/C8	D3/D4	D5/D6	D7/D8	D9/D10
0.33	1.26	1.59	0.42	0.76	1.12	0.54	0.61	0.58

Table S5

sequences 5'-3'	calculated oligonucleotide mass [g/mol]	MALDI-TOF data $[M-H]^-$	sequences 5'-3'	calculated oligonucleotide mass [g/mol]	MALDI-TOF data $[M-H]^-$
CUGUGCACAGTT	3768.4	3761.1	UUGUGCACAUTT	3730.3	3732.4
s2CUGUGCACAGTT	3784.4	3786.9	s2UUGUGCACAUTT	3746.3	3750.6
CUGUGCACAUTT	3729.3	3728.7	AUUUGCAAAUTT	3738.3	3741.2
s2CUGUGCACAUTT	3745.3	3749.9	As2UUUGCAAAUTT	3754.3	3757.7
CUGUGCACAATT	3752.4	3757.0	AUUUGCAAGUTT	3754.3	3755.8
s2CUGUGCACAATT	3768.4	3771.2	As2UUUGCAAGUTT	3770.3	3779.6
CUGUGCACACTT	3728.3	3732.7	AUUUGCAACUTT	3714.3	3715.8
s2CUGUGCACACTT	3744.3	3747.0	As2UUUGCAACUTT	3730.3	3733.4
ACUUGCAAGUTT	3753.3	3748.8	AUUUGCAAUUTT	3715.3	3748.2
As2CUUGCAAGUTT	3769.3	3770.7	As2UUUGCAAUUTT	3731.3	3736.1
ACUUGCAAUUTT	3714.3	3717.8	UAUUGCAAUATT	3738.3	3733.4
As2CUUGCAAUUTT	3730.3	3735.9	UAs2UUGCAAUATT	3754.3	3758.5
ACUUGCAAAUTT	3737.3	3742.0	UAs2UUGCAGUATT	3770.3	3772.6
As2CUUGCAAAUTT	3753.3	3755.8	UAUUGCACUATT	3714.3	3716.8
ACUUGCAACUTT	3713.3	3714.6	UAs2UUGCACUATT	3730.3	3731.8
As2CUUGCAACUTT	3729.3	3731.2	UAUUGCAUUATT	3715.3	3717.6
UACUGCAGUATT	3753.3	3746.0	UAs2UUGCAUUATT	3731.3	3736.4
UAs2CUGCAGUATT	3769.3	3771.9	UUGUGCACAATT	3753.3	3752.9
UACUGCAUUATT	3714.3	3716.9	s4UUGUGCACAATT	3769.3	3773.3
UAs2CUGCAUUATT	3730.3	3732.5	s4UUGUGCACAGTT	3785.3	3789.3
UACUGCAAUATT	3737.3	3741.9	UUGUGCACACTT	3729.3	3734.3
UAs2CUGCAAUATT	3753.3	3756.8	s4UUGUGCACAUTT	3746.3	3748.2

UACUGCACUATT	3713.3	3717.3		AUUUGCAAAUTT	3738.3	3741.2
UAs2CUGCACUATT	3729.3	3734.4		As4UUUGCAAAUTT	3754.3	3759.0
UUGUGCACAATT	3753.3	3752.9		As4UUUGCAAGUTT	3770.3	3773.7
s2UUGUGCACAATT	3769.3	3774.3		AUUUGCAACUTT	3714.3	3719.4
UUGUGCACAGTT	3769.3	3773.1		As4UUUGCAACUTT	3730.3	3733.9
s2UUGUGCACAGTT	3785.3	3787.3		As4UUUGCAAUUTT	3731.3	3734.0
UUGUGCACACTT	3729.3	3734.3		UAs4UUGCACUATT	3730.3	3732.7
s2UUGUGCACACTT	3745.3	3746.5		UAs4UUGCAUUATT	3731.3	3733.6
